# Natural and Experimental Evidence Drives Marmosets for Research on Psychiatric Disorders Related to Stress

**DOI:** 10.3389/fnbeh.2021.674256

**Published:** 2021-06-11

**Authors:** Maria Bernardete Cordeiro de Sousa, Maria Lara Porpino de Meiroz Grilo, Nicole Leite Galvão-Coelho

**Affiliations:** ^1^Brain Institute, Federal University of Rio Grande do Norte (UFRN), Natal, Brazil; ^2^Postgraduation Program in Psychobiology, Federal University of Rio Grande do Norte (UFRN), Natal, Brazil; ^3^Postgraduation Program in Neuroscience, Federal University of Rio Grande do Norte (UFRN), Natal, Brazil; ^4^Laboratory of Advanced Studies in Primates, UFRN-Brazil, and Laboratory of Hormone Measurement, Department of Physiology and Behavior, Natal, Brazil; ^5^Department of Physiology and Behavior, Federal University of Rio Grande do Norte (UFRN), Natal, Brazil; ^6^National Institute of Science and Technology in Translational Medicine, Ribeirao Preto, Brazil

**Keywords:** New World primates, behavior, HPA axis, animal model, depression

## Abstract

Knowledge of the behavioral ecology of marmosets carried out in their natural habitat associated with the advent of a non-invasive technique for measuring steroid hormones in feces has made a significant contribution to understanding their social relationships and sexual strategies. These studies showed that they are mainly monogamous, live in relatively stable social groups according to a social hierarchy in which females compete and males cooperate, and form social bonds similar to humans, which makes this species a potential animal model to study disorders related to social stress. In addition, laboratory studies observed the expression of behaviors similar to those in nature and deepened the descriptions of their social and reproductive strategies. They also characterized their responses to the challenge using behavioral, cognitive, physiological, and genetic approaches that were sexually dimorphic and influenced by age and social context. These findings, added to some advantages which indicate good adaptation to captivity and the benefits of the birth of twins, small size, and life cycle in comparison to primates of the Old World, led to their use as animal models for validating psychiatric diseases such as major depression. Juvenile marmosets have recently been used to develop a depression model and to test a psychedelic brew called Ayahuasca from the Amazon rainforest as an alternative treatment for major depression, for which positive results have been found which encourage further studies in adolescents. Therefore, we will review the experimental evidence obtained so far and discuss the extension of the marmoset as an animal model for depression.

## Introduction

Several species and numerous protocols have been tested to validate translational animal models which better mimic the physiological and behavioral states observed in human psychopathologies associated with chronic social stress including fish species (Øverli et al., 2004; Schjolden et al., 2005), birds (Roberts et al., 2007), rodents (Sgoifo et al., 1999; Veenema et al., 2003; Kompagne et al., 2008; Martinez et al., 2008; Hennessy et al., 2009), sheep (Stackpole et al., 2006), pigs (van der Staay et al., 2008), and non-human primates (Barros and Tomaz, 2002; Dettling et al., 2007).

These models show mammals present great genetic similarities and share corresponding neurophysiological mechanisms involved in socialization. However, their social organizations reveal a large range of differences in their size, composition, social bonding, parental care, and reproductive skew, creating a diversity of societies which is currently under investigation in terms of studying their complexity. This evidence demonstrates that the evolutionary pressures related to the natural environment demand adaptive responses which lead to a great diversity of strategies, thus making it difficult to apply the findings of some animal models to people. Therefore, these interspecific differences limit the suitability of a given species as an experimental model for comparative studies with humans (Kappeler, 2019), including non-human primates.

The common marmoset is a small New World primate and has gained attention mainly due to its complex social organization. This species has several social behaviors which are linked to auditory and visual cues like humans, including that they show gazing behavior, intentional pro-social behavior (Burkart and van Schaik, 2020; also known as altruism in humans), and observational social learning. In fact, their social cognition and communication seem more similar to that of humans than those of rhesus monkeys (Burkart and Finkenwirth, 2015). It is also suggested that humans and marmosets share main traits as cooperative breeders such as social tolerance and social monitoring resulting from cooperative breeding (Burkart and Finkenwirth, 2015).

Marmosets exhibit a range of changes at physiological and behavioral levels when facing stressful situations which depends on the individual, social and environmental characteristics resembling those observed in humans. They also have a well-defined ethogram (Stevenson and Poole, 1976), and there are procedures to measure cortisol in blood and urine, a non-invasive technique using feces (see de Sousa, 2017), and new methods have more recently been standardized for saliva (Cross and Rogers, 2004; Cross et al., 2004; Ash et al., 2018) in the laboratory and in hair in captive (Phillips et al., 2018) and wild marmosets at different ages (Garber et al., 2020).

These features make this species relevant for their use as a model in psychiatry studies related to chronic stress. It is largely understood that chronic social stress is the main etiology of Major Depressive Disorder (MDD; Tafet and Bernardini, 2003), General Anxiety Disorder (GAD; Juruena et al., 2020), and Post-Traumatic Stress Disorder (PTSD; Yehuda et al., 2015). In this sense, the cortisol profile is considered the marker of hypothalamus-pituitary-adrenal (HPA) axis disruption and has been used as a potential biomarker of some psychiatric syndromes (Dieleman et al., 2015; Herman et al., 2016). Thus, in this minireview, we aim to analyze recent studies to provide more evidence for the use of common marmosets as one of the strongest candidates today as an experimental model for research on psychiatric disorders related to stress.

## Behavioral and Hypothalamic-Pituitary-Adrenal Axis Characterization in Common Marmosets

Despite there being several protocols for inducing psychiatric disorders in animal models such as genetic (Sasaki, 2015), pharmacological (Costall et al., 2011; Pryce et al., 2011; Yasue et al., 2015), and behavioral (Cilia and Piper, 1997; Barros et al., 2000, 2008; Dettling et al., 2002b), the etiological models for mental disorders associated to chronic stress based on social challenging are closer to that which triggers human PTSD (Dettling et al., 2002a,b, 2007), GAD (Cilia and Piper, 1997; Barros et al., 2000, 2008) and MDD (Johnson et al., 1996; Galvão-Coelho et al., 2008, 2017). Therefore, animal models are promising to gain larger emphasis in scientific studies. In order to do so, it is essential to investigate and understand the neurobiological mechanisms underlying the associated physiological and psychological mechanisms that support the analysis of psychiatric disorders. The data obtained in studies using non-human models have significant limitations because the etiology of psychiatric disorders is characterized by a multiplicity of causal factors and symptoms (Salgado and Sandner, 2013). Furthermore, in the perspective that it is the combination of “nature” and “nurture” which contribute to different phenotypes of susceptibility and resilience to developing mood disorders (Hollander et al., 2020), the need for substantial information to attain the validation criteria of an animal model is strengthened.

The systematic study of the behavior and development of common marmosets (*Callithrix jacchus*) living in captivity in colonies worldwide started in the 1970s (Epple, 1970; Abbott and Hearn, 1978), and wild groups started to be monitored in Brazil in the 1980s (Stevenson and Rylands, 1988). In this context, the common marmoset is currently one of the most studied species among non-human primates in both wild and in captivity situations and consequently, the marmoset emerges as a distinct experimental model with respect to the amount of information available in both settings.

### Natural Environmental Studies

Common marmosets are small New World primates of the Callitrichidae family. This species is not threatened and easily found in Brazilian Atlantic Forest, usually living in stable social groups composed from three to 19 familiar individuals (Stevenson and Rylands, 1988; Digby, 1995; Schiel and Souto, 2017). The group composition varies depending on the number of births, migrations, and disappearances (Digby and Barreto, 1993). Studies also show that they are cooperative breeders, suggesting extended family groups in which reproductive pairs (dominants) develop special social bonds, present parental and alloparental care more frequently involving older siblings and close relatives (Digby, 1995). Despite groups generally being composed of relatives, unrelated members have also been identified in the social group (Faulkes et al., 2009).

The core of the social group is the monogamous breeding couple, which has very low sexual body dimorphism, but polygynous groups have also been described (Digby, 1999; Arruda et al., 2005). It is interesting to note that marmoset males are more cooperative and build stronger intra-sexual ties, while female marmosets exhibit higher intra-sexual competition levels (Arruda et al., 2005; Yamamoto et al., 2010; de Sousa et al., 2019).

The intra-group organization of marmosets can be disturbed during social reproduction dynamics, as shown in situations where dominant females perpetrated infanticide toward the offspring of subordinates (Digby, 1995; Arruda et al., 2005) or during the subordinate scaping of ovulatory inhibition (Albuquerque et al., 2001), as well as when males were facing territorial dispute challenges (Digby, 1995; Lazaro-Perea, 2001), thus producing tensions and activating the stress response system (de Sousa et al., 2019). Furthermore, both behavioral repertoire (episodes of aggression and open fights expressed by piloerection displays, scent marking, and anogenital presentation) and associated endocrine correlates increased cortisol in females and cortisol and androgens in males during these situations, being characteristic of the stress response of the animals when exposed to natural stressors. These reactions characterize the typical stress response that is shared by mammals, including humans.

### Laboratory Studies

The first studies developed in common marmosets under captive conditions date from the 1970s throughout the 1980s and described data related to marmosets’ behavior living in colonies, such as: living in family groups (Epple, 1970), breeding and development (Abbott and Hearn, 1978; Tardif et al., 2003), parental care (Ingram, 1977), first cohabitation weeks of common marmoset heterosexual pairs (Evans and Poole, 1983; Woodcock, 1983), and the construction of an ethogram of social behavior in different contexts (piloerection displays, adult play, copulatory, aggressive, and prey-catching behavior and vocalization; Stevenson and Poole, 1976). These studies were fundamental to provide information to researchers to learn about and better understand the role of their behaviors and how they vary along with their age, as well as the endocrine profile in the transition phases of puberty (Abbott and Hearn, 1978), first ovulation and initial age for pregnancy (Tardif et al., 2003). Three classifications were proposed to characterize the development stages of the common marmoset (Missler et al., 1992; Yamamoto, 1993; de Castro Leão et al., 2009), and a comparative analysis was carried out (Schultz-Darken et al., 2016) providing a reference scale for long-term studies.

A longitudinal study by our laboratory on the developmental stages of common marmosets from the infant stage (3 months) to the sub-adult (15 months) describes the cortisol variation in males and females in relation to age and sex. Cortisol changes throughout the marmoset’s life stages were recorded, with the highest cortisol levels being observed in the infant phase and tending to decrease with development, especially in males (de Sousa et al., 2015). A better understanding of marmoset development allows appropriate parameters to be available to compare the normal and abnormal development after birth and to assist in psychiatric studies of early life chronic stress (Dettling et al., 2002a,b, 2007; Pryce et al., 2004a,b, 2011; Arabadzisz et al., 2010) and to assess neurodevelopment from the perspective of using biomedical research in transgenic approaches (Sasaki et al., 2009).

Sexual dimorphism is observed from the juvenile stage at baseline cortisol levels in favor of female marmosets, which have higher fecal cortisol levels than males (de Sousa et al., 2015). However, despite the sex, it is suggested that the marmoset’s temperament also modulates basal cortisol levels, with three distinct basal cortisol profiles being described in adult marmosets of both sexes scaled as low, medium, and high (Galvão-Coelho et al., 2008).

Moreover, a sexual dimorphic stress response was evidenced by distinct cortisol increases when adult marmosets were moved to new cages, which varies depending on the type of stressor, the presence or not of a co-specific, and the type of social support (Galvão-Coelho et al., 2012; de Menezes Galvão et al., 2016). Dyad formation also triggered a sexual dimorphic stress response in adult marmosets. Females were more reactive than males when facing a pair formation as well as in response to isosexual dyad formation, independent of the co-specific being familiar or not (Galvão-Coelho et al., 2012). Adult male marmosets were also more sensitive to social isolation, showing larger cortisol increases (Castro and de Sousa, 2005).

Other convergent data in terms of sexual dimorphism in the stress response in marmosets was evidenced in a genetic study where the NTRK2, CREB1, and CRTC1 genes possibly associated with CREB1-BDNF-NTRK2 pathway have a higher expression level in females, suggesting greater demand for this signaling cascade in females (Nogueira et al., 2018). This pathway presents an important role in brain adaptation to stress and variations in these genes have been identified as probable risk factors for depression in humans (Juhasz et al., 2011).

Stress and psychiatric studies are usually based on male models. However, there is a long-standing demand for psychiatric models which include both sexes (Kokras and Dalla, 2014; Alshammari, 2021). Therefore, sexual dimorphism in the HPA axis and in the autonomic component of the stress response in marmosets are important characteristics of this species which resemble humans and enables its use in sex-related psychopathology studies. It is assumed that better treatments can be validated if both sexes are included as pre-clinical models of psychiatric diseases (Dalla et al., 2010).

Laboratory studies also demonstrated a diurnal (de Sousa and Ziegler, 1998; Ferreira Raminelli et al., 2001) and annual fluctuation in plasma cortisol in common marmosets over a 2-year interval in which a marginal effect of sex was observed with females showing higher levels during dry seasons (Cunha et al., 2007). The annual variation in cortisol was associated with precipitation and temperature, but not with the photoperiod, due to the small variation in the photophase length at the Equator line where our animals live. Therefore, the circadian and seasonal change in cortisol can also contribute to approaches which explore some characteristics in physiological regulation using appropriate protocols which may be relevant for models to investigate circadian changes in the cortisol profile of depressed patients (Keller et al., 2006) and for seasonal depression (Thalén et al., 1997).

## The Common Marmoset as An Animal Model to Study Psychopathologies Associated with Chronic Stress

Despite recognition of increasing psychiatric disorder incidences being related to chronic stress in adolescents and the importance of chronic stress in this ontogenetic phase on long–term non-adaptive neuroplasticity changes (Thapar et al., 2012; Knowles et al., 2021), a large part of the literature about chronic stress related to neuropsychiatric disorders using marmoset models have only explored adult (Johnson et al., 1996; Cilia and Piper, 1997; Barros et al., 2000, 2008) and infant (Dettling et al., 2002a,b, 2007) ages. Therefore, being aware of this gap, our group recently validated the marmoset as a juvenile animal model for major depression (Galvão-Coelho et al., 2017).

In aiming at the complete validation of this juvenile marmoset animal model for major depression, we developed a protocol addressing: (a) ecological validity, which means that the symptoms in this model were triggered by similar occurrences for human diseases, and in this case we used the social isolation protocol; (b) construct validity: animals showed activation of physiological processes in a similar way to humans, such as cortisol levels; (c) face validity: the animals expressed symptoms/behavior changes similar to those of humans, such as changes in appetite and anhedonia; (d) predictive validity: animals and humans showed similar responses to treatments; (e) interrelational validity: we investigated the model in several domains such as behaviors, molecular biomarkers and cognition; (f) evolutionary criteria: reflects the ability of the proposed model to investigate specific domains in various species such as anhedonia, body weight and cortisol analysis, which can be done on marmosets, humans and other species such as rodents; and (g) population validity: the capacity of the proposed model to reflect the natural variance of phenotypes observed in the general population, as observed in some studies in the literature (Galvão-Coelho et al., 2008, 2017).

In this context, male and female juvenile marmosets (approximately 7 months) were subjected to a chronic social stressor protocol (13 weeks of social isolation) to induce a state of depression. Next, fecal cortisol, body weight, and some intra and interspecific behaviors were analyzed in the stressed group and compared with a control group where marmosets continued to live with the family throughout the study. In short, we observed a behavioral and neuroendocrine depressive state similar to patients with depression (Dean and Keshavan, 2017; Santiago et al., 2020) and other non-human primate models of depression (Li et al., 2013; Hennessy et al., 2014) for both sexes isolated from marmosets (male; female), but not for animals kept with the family. Symptoms included a significant reduction in cortisol levels, an increase in anxiety-like behavior (stereotypes, such as scratching and body piloerection), presence of anhedonia, sexual dimorphic changes in diet and body weight (Galvão-Coelho et al., 2017). Most of the altered behaviors, such as scratching, as well as the change in cortisol levels, were reversed by treatment with nortriptyline (7 days), but not by the vehicle (Galvão-Coelho et al., 2017).

The validation of this protocol gave way to developing depressive juvenile models with other non-human primate species (Teng et al., 2021) and enabled a step forward when a potential new alternative drug therapy (nortriptyline) for depression was tested (da Silva et al., 2019). Using that same validated protocol, some male and female juvenile marmosets (male; female) were treated with a single dose of ayahuasca (da Silva et al., 2019). Ayahuasca is a psychedelic decoction made from combining two plants from the Amazon rainforest: *Psychotria viridis* and *Banisteriopsis caapi*, which has shown promising fast antidepressant effects in humans (Palhano-Fontes et al., 2014, 2021; Perkins et al., 2021; Sarris et al., 2021)*.* Thus, we observed a fast recovery in the decreased cortisol levels (hypocortisolemic state) and the bodyweight of depressive-like marmosets to the baseline values 24 h after an acute dose of Ayahuasca, and not after the vehicle, as well as reductions in anxiety-like stereotypic behaviors. Additionally, the single dose of ayahuasca also had long-lasting effects (14 days) and seemed to be more promising than nortriptyline treatment (da Silva et al., 2019).

These findings in the marmoset supported the development of clinical trials using ayahuasca treatment for resistant depression and showed positive results, including some similar to those seen in the marmoset (for example, regulating cortisol levels; de Almeida et al., 2019; Palhano-Fontes et al., 2019; Galvão-Coelho et al., 2020, 2021).

## Future Directions

As mentioned earlier in this review, some scientists argue that current animal models are insufficient to express the full extent of mood disorders and therefore have no real value in understanding mental illness. However, new research initiatives to integrate the effort to study mental disorders, such as the Roadmap for Mental Health Research in Europe (ROAMER; Haro et al., 2014) and Research Domain Criteria (RDoC), funded by European and American public and private institutions (Insel et al., 2010; Elfeddali et al., 2014) intend to build a matrix of variables which constitute a unit of analysis involving studies from genetic, physiological, behavioral, and self-report evaluations. New information is added or contributions are made in these projects by revision of earlier knowledge on psychopathology analysis of symptoms, and both initiatives will facilitate progress in this field. They argue that these disorders are too complex to try to understand globally, so the knowledge gained step-by-step should provide more clarity and effectiveness. In addition, some specific symptoms are common to different psychopathologies, so that understanding one symptom for a certain disorder can be applied to another. Therefore, this new understanding makes studies on animal models even more important, since it is easier to focus on fewer symptoms and their pathophysiological correlates and their management. In turn, encouraging the development of new protocols and stimulating new studies on biomarkers, as well as on the development of new drugs. In addition, marmosets are the focus of a large–scale Japanese project called Brain Mapping by Integrated Neurotechnologies for Disease Studies (Brain/MINDS; Okano and Mitra, 2015; Okano et al., 2015), and new colonies of marmosets show that the use of this small neotropical primate as experimental models continues to present a promising future for scientific research, including for investigations of neuropsychiatric diseases.

## Author Contributions

MS and NG-C wrote and edited the manuscript. MS, NG-C, and MM performed bibliographic research and main drafting. All authors contributed to the article and approved the submitted version.

## Conflict of Interest

The authors declare that the research was conducted in the absence of any commercial or financial relationships that could be construed as a potential conflict of interest.
